# Adolescent multi-omics and Mendelian randomization reveal transdiagnostic molecular mechanisms in psychiatric disorders

**DOI:** 10.1038/s44220-026-00660-2

**Published:** 2026-06-05

**Authors:** Luheng Qian, Runye Shi, Xinyang Yu, Di Chen, Tobias Banaschewski, Arun L. W. Bokde, Herta Flor, Antoine Grigis, Hugh Garavan, Penny Gowland, Andreas Heinz, Jean-Luc Martinot, Marie-Laure Paillère Martinot, Eric Artiges, Frauke Nees, Dimitri Papadopoulos Orfanos, Luise Poustka, Sarah Hohmann, Nathalie Holz, Michael N. Smolka, Nilakshi Vaidya, Henrik Walter, Robert Whelan, Gunter Schumann, Xiaolei Lin, Sylvane Desrivières, IMAGEN Consortium, Tobias Banaschewski, Arun L. W. Bokde, Herta Flor, Antoine Grigis, Hugh Garavan, Penny Gowland, Andreas Heinz, Jean-Luc Martinot, Marie-Laure Paillère Martinot, Eric Artiges, Frauke Nees, Dimitri Papadopoulos Orfanos, Luise Poustka, Sarah Hohmann, Nathalie Holz, Michael N. Smolka, Nilakshi Vaidya, Henrik Walter, Robert Whelan, Gunter Schumann, Sylvane Desrivières

**Affiliations:** 1 School of Data Science, Fudan University, Shanghai, China; 2 Social, Genetic and Developmental Psychiatry Centre, Institute of Psychiatry, Psychology and Neuroscience, King’s College London, London, UK; 3 Institute of Science and Technology for Brain-Inspired Intelligence, Fudan University, Shanghai, China; 4 Department of Child and Adolescent Psychiatry and Psychotherapy, Central Institute of Mental Health, Medical Faculty Mannheim, Heidelberg University, Mannheim, Germany; 5 German Center for Mental Health (DZPG), Mannheim, Germany; 6 Discipline of Psychiatry, School of Medicine and Trinity College Institute of Neuroscience, Trinity College Dublin, Dublin, Ireland; 7 Institute of Cognitive and Clinical Neuroscience, Central Institute of Mental Health, Medical Faculty Mannheim, Heidelberg University, Mannheim, Germany; 8 Department of Psychology, School of Social Sciences, University of Mannheim, Mannheim, Germany; 9 NeuroSpin, CEA, Université Paris-Saclay, Gif-sur-Yvette, France; 10 Department of Psychiatry and Department of Psychology, University of Vermont, Burlington, VT USA; 11 Sir Peter Mansfield Imaging Centre School of Physics and Astronomy, University of Nottingham, Nottingham, UK; 12 Department of Psychiatry and Psychotherapy, University of Tübingen, Tübingen, Germany; 13 German Center for Mental Health (DZPG), Tübingen, Germany; 14 INSERM U1299 ‘Developmental Trajectories in Psychiatry and Addictology’, University Paris-Saclay, Ecole Normale Supérieure Paris-Saclay, Mathematics Department, Centre Borelli CNRS, Gif-sur-Yvette, France; 15 Department of Psychiatry, LabD-Psy, Barthélémy Durand Hospital, Etampes, France; 16 Department of Child and Adolescent Psychiatry, AP-HP, Sorbonne Université, Pitié-Salpêtrière Hospital, Paris, France; 17 Institute of Medical Psychology, Ludwig-Maximilians-Universität (LMU) in Munich, Munich, Germany; 18 Department of Child and Adolescent Psychiatry, Center for Psychosocial Medicine, University Hospital Heidelberg, Heidelberg, Germany; 19 Department of Child and Adolescent Psychiatry and Psychotherapy, University Medical Center Hamburg-Eppendorf, Hamburg, Germany; 20 Department of Psychiatry and Neuroimaging Center, Technische Universität Dresden, Dresden, Germany; 21 Centre for Population Neuroscience and Stratified Medicine (PONS), Department of Psychiatry and Psychotherapy, Charité Universitätsmedizin Berlin, Berlin, Germany; 22 Department of Psychiatry and Psychotherapy CCM, Charité – Universitätsmedizin Berlin, corporate member of Freie Universität Berlin, Humboldt-Universität zu Berlin and Berlin Institute of Health, Berlin, Germany; 23 School of Psychology and Global Brain Health Institute, Trinity College Dublin, Dublin, Ireland; 24 Centre for Population Neuroscience and Precision Medicine (PONS), Institute for Science and Technology of Brain-inspired Intelligence (ISTBI), Fudan University, Shanghai, China; 25 Huashan Institute of Medicine, Huashan Hospital affiliated to Fudan University, Shanghai, China

**Keywords:** DNA methylation, Gene expression, Psychiatric disorders

## Abstract

The biological mechanisms underlying major neuropsychiatric disorders remain largely elusive. Given the frequent association of immune dysregulation with these conditions, we used blood-derived multi-omics data from 1,274 healthy adolescents in the IMAGEN cohort to identify transdiagnostic biomarkers and mechanisms that could inform diagnosis and treatment. We first conducted genome-wide analyses to identify single nucleotide polymorphisms associated with DNA methylation and gene expression, with findings replicated in external datasets. These quantitative trait loci were further explored through Mendelian randomization analyses across 6 neuropsychiatric disorders (attention deficit hyperactivity disorder, autism spectrum disorder, bipolar disorder, major depressive disorder, schizophrenia and insomnia), leading to the identification of 73 putatively causal CpG sites and 62 genes that were either unique to individual disorders or, as in the case of *MRPL2*, shared among the disorders. Notably, the identified genes were significantly enriched in pathways linked to both psychiatric and autoimmune diseases, suggesting a shared genomic architecture between autoimmune and neuropsychiatric disorders. Two-step Mendelian randomization and colocalization analyses revealed potential transdiagnostic regulatory pathways, in which the expression of three genes (*MAD1L1*, *MRPL2* and *HLA-DRB1*) mediated the effects of CpG methylation on schizophrenia and insomnia. Specifically, DNA methylation at cg06770790 repressed *MRPL2*, which was putatively causal for insomnia (*β* = −0.38, *P* = 1.29 × 10^−4^) and schizophrenia (*β* = −0.38, *P* = 1 × 10^−4^). Conversely, increased expression of *MAD1L1* and *HLA-DRB1*, driven by methylation at several CpG sites, was potentially causal for schizophrenia. Our findings highlight key molecular mechanisms and genes implicated in neuropsychiatric disorders, offering promising new targets for therapeutic intervention.

## Abstract (web summary)

This research utilized multi-omics data from 1,274 adolescents to identify transdiagnostic biomarkers for neuropsychiatric disorders, employing genome-wide analyses and Mendelian randomization to uncover causal pathways linking immune dysregulation to psychiatric conditions and revealing potential therapeutic targets.

## Main

Approximately one in eight people worldwide live with a mental disorder, and this number continues to rise^1^. According to studies by the Global Burden of Disease, neuropsychiatric disorders are among the leading causes of disability and are important risk factors for premature mortality^2^. These disorders have profound psychological and psychiatric effects on the workforce and impose a substantial economic burden on society^3^. Neuropsychiatric disorders such as schizophrenia (SCZ), major depressive disorder (MDD), bipolar disorder (BIP), autism spectrum disorder (ASD) and attention deficit hyperactivity disorder (ADHD) are characterized by their moderate heritability and peak age at onset in childhood or adolescence^4^. Despite this, the underlying biological mechanisms remain poorly understood, limiting progress in early detection and effective treatment.

Twin studies have provided empirical evidence of the genetic contributions to neuropsychiatric disorders^5–8^ and genome-wide association studies (GWAS) have identified hundreds of genetic loci associated with multiple neuropsychiatric disorders^9–15^. However, most associated variants lie in non-coding regions^16,17^, making it difficult to infer their regulatory roles or downstream biological effects. Genetic variants associated with neuropsychiatric disorders may influence disease risk through regulatory mechanisms, including effects on DNA methylation and gene expression, captured by methylation quantitative trait loci (meQTLs) and expression quantitative trait loci (eQTLs). Integrative approaches that combine genetic and functional genomic data can help bridge this gap by mapping genetic risk to molecular mechanisms. Mendelian randomization (MR) further enables the inference of putative causal relationships between these molecular intermediates and disease outcomes, leveraging genetic instruments to move beyond correlation toward causal insight.

Although neuropsychiatric disorders primarily affect the brain, increasing evidence suggests that peripheral tissues, especially blood, can provide meaningful molecular insights into psychiatric risk. This is particularly relevant for disorders with known immune involvement, such as SCZ, MDD, BIP, ASD and ADHD^18–28^. Multiple studies have reported systemic inflammation and immune dysregulation in these disorders, and GWAS have consistently implicated immune-related pathways, especially in SCZ and MDD^9,29^. Blood-based methylation and gene expression profiles can reflect such systemic immune alterations and may capture upstream regulatory mechanisms that affect both peripheral and central systems^30^. Moreover, blood-derived molecular QTLs are highly accessible and scalable for population-based research, particularly in adolescents, where brain tissue is not available. While blood may not fully represent brain-specific mechanisms, it offers a valuable proxy for identifying peripheral biomarkers and genetically anchored regulatory pathways with potential relevance to neurodevelopmental and psychiatric conditions^31^.

However, existing multi-omics studies in psychiatry have been limited by a focus on adult samples, single disorders or correlational designs. This has left a significant gap in understanding how early molecular regulatory mechanisms may contribute to shared and distinct psychiatric risk across disorders during adolescence, the developmental window when many of these conditions first emerge.

**Fig. 1:**
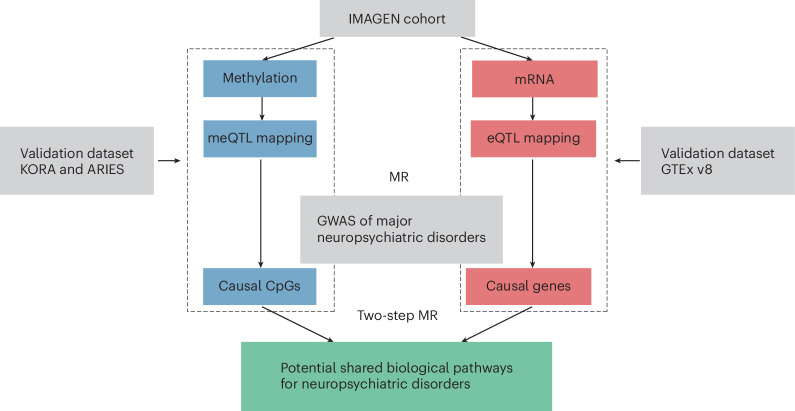
Study design. Schematic workflow to investigate the shared regulatory pathways across major neuropsychiatric disorders through integration of genomic, epigenomic and transcriptomic profiles.

In this study, we address this gap by generating and analyzing a multi-omic QTL resource in adolescents and uncover putative causal mechanisms underlying neuropsychiatric disorders. We performed genome-wide analyses of meQTLs and eQTLs in blood samples from 14-year-old participants in the IMAGEN cohort, with replication in KORA^32^, ARIES^33^ and GTEx v8 (ref. ^34^). Using MR and colocalization analyses, we integrated these QTLs with GWAS summary statistics for six neuropsychiatric disorders: ADHD, ASD, BIP, MDD, SCZ and insomnia (INS). Our goals were to (1) identify CpG sites and genes with genetically informed causal associations to psychiatric risk during adolescence, (2) assess which features are transdiagnostic versus disorder specific, and (3) apply two-step MR to explore regulatory pathways linking DNA methylation to gene expression and disease risk. These findings provide new insights into adolescent molecular mechanisms of psychiatric disorders and potential transdiagnostic targets for early intervention. Figure 1 illustrates the study design.

## Results

### Description of the IMAGEN cohort

The IMAGEN study is a multicenter longitudinal adolescent cohort that aims to explore the genetic, behavioral and neurobiological underpinnings of individual differences for neurocognition and neurodevelopment^35^. Approximately 2,000 healthy adolescents, aged 14 at baseline, were recruited across 8 European sites (Berlin, Dresden, Dublin, Hamburg, London, Mannheim, Nottingham and Paris), with follow-up visits at 16, 19 and 23 years old. Our study focuses on the multi-omics data collected at baseline. Genotype, blood-derived DNA methylation and RNA expression data were available for 1,324, 1,274 and 545 participants at baseline, respectively.

### Genome-wide identification and validation of *cis*-meQTLs

Pairwise genome-wide associations between 5.9 million single nucleotide polymorphisms (SNPs) and DNA methylation at 372,582 CpG sites from 1,274 IMAGEN participants were analyzed to identify *cis*-meQTLs^36^, defined as SNPs significantly associated with DNA methylation, and residing within a 2-megabase (Mb) window of the associated CpG site. After Bonferroni correction (that is, *P* < 3.1 × 10^−11^ for 1.6 × 10^9^ *cis*-SNP–CpG pair associations), a total of 9,105,155 *cis*-meQTL–CpG pairs (comprising 2,196,765 *cis*-meQTLs and 58,525 CpG sites) were identified.

**Fig. 2:**
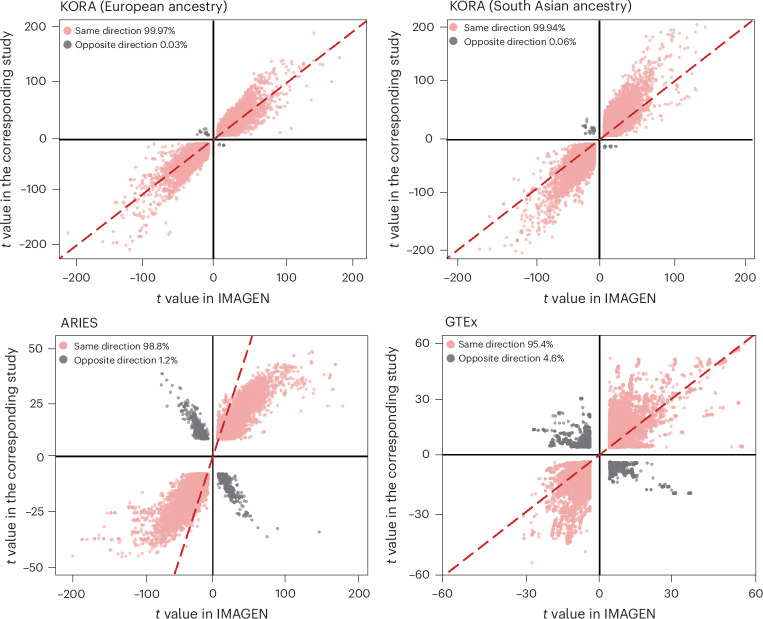
Validation of *cis*-meQTLs and *cis*-eQTLs identified in IMAGEN via external studies. *t* values of *cis*-meQTLs identified in IMAGEN were plotted against those from KORA (European ancestry (top left) and South Asian ancestry (top right)) and ARIES (adolescents of European ancestry; bottom left). *t* values of *cis*-eQTLs identified in IMAGEN were plotted against those from GTEx (bottom right). Points in red indicate the same effect direction between SNP–CpG and SNP–mRNA pairs. The 45° dashed line indicates identical *t* values in IMAGEN and the corresponding validation cohort. A total of 100,000 SNP–CpG and SNP–mRNA pairs were randomly selected for better visualization.

These findings were replicated (Bonferroni-corrected *P* < 1 × 10^−14^) in 2 independent datasets: KORA, a study with participants of European and South Asian ancestries^32^, and ARIES, an adolescent cohort^33^. In KORA, 32.49% (*N* = 2,958,057) and 39.77% (*N* = 3,621,238) of the *cis*-meQTL–CpG pairs were replicated when using samples from the same ancestry (that is, European) and from South Asian ancestry, respectively. Allelic effects were highly concordant among these replicated *cis*-meQTLs, with concordance observed for 99.97% (*N* = 2,957,003) and 99.94% (*N* = 3,619,041) of the replicated *cis*-meQTLs in the European and South Asian samples, respectively (Fig. 2a,b). Correlations of *t* values were also extremely high (*r* = 0.95 and 0.93 in the European and South Asian sample, respectively). In ARIES, 15.20% (*N* = 1,383,576) of the identified *cis*-meQTL–CpG pairs were replicated, among which 98.8% (*N* = 1,366,292) concorded for direction of effect (Fig. 2c). Again, the correlation of *t* values in this comparison was remarkably high (*r* = 0.94).

### Genome-wide identification and validation of *cis*-eQTLs

Pairwise association between 5.9 million SNPs and 19,840 mRNA expression profiles from 545 IMAGEN adolescents were used to map *cis*-eQTLs^34^ within a 2-Mb window. A total of 358,616 *cis*-eQTL–mRNA pairs (comprising 280,161 *cis*-eQTLs and 4,524 mRNAs) were identified (false discovery rate (FDR)-corrected *P* value (*P*_FDR_) < 0.05). Of these, 48.26% (*N* = 173,081) were replicated (*P*_FDR_ < 0.05) in the GTEx v8 dataset^34^. Directions of effects were highly consistent for 95.36% (*N* = 165,051) of the replicated *cis*-eQTL–mRNA pairs (correlation of *t* values = 0.80; Fig. 2d).

### Identification of putatively causal CpGs for neuropsychiatric disorders

We next used two-sample MR analyses to identify putatively causal (hereafter named as causal) CpGs for neuropsychiatric disorders. Disorders used as outcome measures included ADHD, ASD, BIP, MDD, SCZ and INS.

**Fig. 3:**
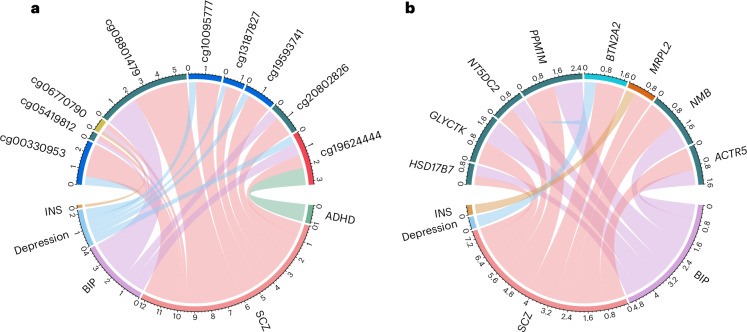
Causal CpGs and causal genes shared by major neuropsychiatric disorders. **a**,**b**, The causal CpGs (**a**) and causal genes (**b**) are shown. Each neuropsychiatric disorder is represented by a specific color, and the width of each curve represents the effect size of the causal CpGs or genes on the corresponding disorder (wider curves indicate larger absolute effect sizes). Numbers indicate correlations between corresponding CPG/gene and neuropsychiatric disorder.

**Fig. 4:**
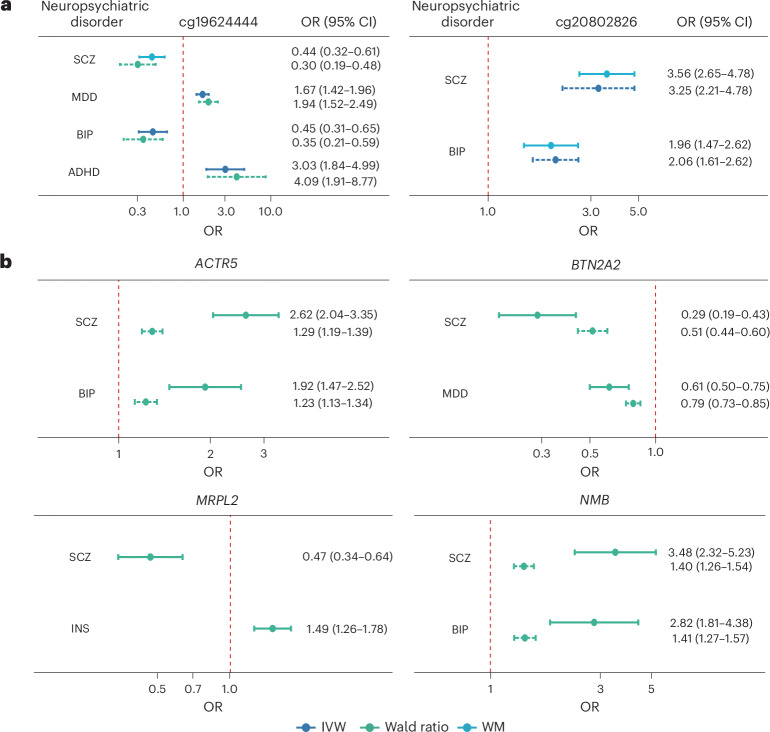
Causal CpGs and causal genes shared between multiple neuropsychiatric disorders. **a**,**b**, Two-sample MR was used to identify causal CpGs (**a**) and causal genes (**b**) for each neuropsychiatric disorder based on the GWAS summary statistic (*n*_eff_ = 128,214 for ADHD, *n*_eff_ = 389,523 for BIP, *n*_eff_ = 1,816,976 for MDD, *n*_eff_ = 313,750 for INS and *n*_eff_ = 233,471 for SCZ). eff, effective. Solid lines represent causal estimates using *cis*-meQTLs (*n* = 1,274) and *cis*-eQTLs (*n* = 545) identified in IMAGEN as the genetic instruments, and the dashed line represents the replicated estimates using *cis*-meQTLs from KORA European ancestry (*n* = 3,799) datasets and *cis*-eQTLs from GTEx (*n* = 488), with different MR methods (indicated by colors) adopted as appropriate. The error bar indicates the 95% confidence interval of the OR estimates, derived from the s.e. of the MR effect estimate.

First, to derive valid instrumental variables (IVs), all *cis*-meQTLs identified in IMAGEN were clumped using a linkage disequilibrium (LD) threshold of *r*^2^ < 0.01 within a 2-Mb window, resulting in 10,986 independent *cis*-meQTLs (associated with 7,669 CpGs). Next, these independent *cis*-meQTLs were associated to GWAS summary statistics for ADHD, ASD, BIP, SCZ, MDD and INS (Supplementary Table 1). For each disorder, CpGs associated with at least 3 independent *cis*-meQTLs were selected as exposure variables in the MR analyses (1,267 candidate CpGs for ADHD, 1,509 for ASD, 1,324 for BIP and SCZ, 1,131 for MDD and 1,233 for INS) and independent *cis*-meQTLs were used as genetic instruments. After Bonferroni correction, a total of 73 unique causal CpGs were identified (Supplementary Table 2a). These included 3 CpGs for ADHD, 1 for ASD, 9 for BIP, 58 for SCZ, 12 for MDD and 2 for INS. Nine CpGs were causal for more than one neuropsychiatric disorder (Fig. 3). Notably, cg19624444 was simultaneously causal for ADHD, BIP, SCZ and MDD. This CpG is located within *MAD1L1*, whose differential DNA methylation was previously found to be associated with SCZ^37^ and phenotypes of depression^38^. Intriguingly, the direction of effects for cg19624444 methylation differed across disorders, with hypermethylation associated with ADHD (odds ratio (OR) = 3.03, *P* = 1 × 10^−5^) and MDD (OR = 1.67, *P* = 4 × 10^−10^) and hypomethylation associated with BIP (OR = 0.45, *P* = 2 × 10^−5^) and SCZ (OR = 0.44, *P* = 1 × 10^−6^) (Fig. 4a). All other CpGs causal for multiple disorders were located on chromosome 6. These included four CpGs (cg00330953, cg13187827, cg19593741 and cg10095777) within the *SAPCD1-AS1*/*VWA7* gene region, causal for both SCZ and MDD, and three CpGs (cg20802826, cg05419812 and cg08801479) causal for both SCZ and BIP.

To replicate these findings, we repeated the MR analysis using published meQTL summary statistics of European ancestry in KORA^32^. A total of 56 causal CpGs were replicated, among which 35 CpGs (62.5%) showed consistent effect directions. In addition, 29 (51.8%) CpGs achieved smaller *P* values after meta-analyzing with the discovery MR, supporting their high reliability.

Comparisons of DNA methylation in blood and brain tissues (3 cortical regions and cerebellum) sampled from the same individuals revealed that, for 64% of the replicated CpGs, correlations between blood and brain were significant (Supplementary Table 2b). These were positively correlated, except for cg19624444, the CpG causal for ADHD, BIP, SCZ and MDD, for which correlations between blood and brain (that is, prefrontal cortex, superior temporal cortex and cerebellum) were negative (*r* = −0.49 to −0.58).

### Identification of putatively causal genes for neuropsychiatric disorders

To investigate differences in gene expression as potential mechanisms underlying SNP–disease associations in neuropsychiatric disorders, we again used MR analysis. Following clumping using *r*^2^ < 0.01 as the LD threshold, 9,579 *cis*-eQTLs (associated with 6,864 genes) were identified in the IMAGEN cohort and used as the genetic instruments. Those *cis*-eQTLs overlapping with the GWAS summary statistics for each disorder were then taken forward for each MR analysis, resulting in approximately 4,000 differentially expressed genes (3,974 genes for ADHD, 3,864 genes for ASD, 4,186 genes for BIP, 4,186 genes for SCZ, 4,190 genes for MDD and 3,838 genes for INS) being analyzed. After Bonferroni correction for multiple testing, a total of 62 unique genes were identified as putatively causal for at least 1 neuropsychiatric disorder (that is, 2 causal genes for ADHD, 1 for ASD, 16 for BIP, 7 for MDD, 1 for INS and 43 for SCZ) based on their expression levels in blood (Supplementary Table 3a). Among these, eight were causal for more than one neuropsychiatric disorder.

We leveraged eQTLs identified from the GTEx v8 dataset to replicate these findings. A total of 47 causal genes were replicated when considering blood eQTLs, among which 39 (82.3%) showed consistent effect directions with the discovery sample, and 25 (53.2%) achieved smaller *P* values after meta-analyzing with the discovery MR (Fig. 3 and Supplementary Table 3a). Figure 4b shows the replicated causal genes that share their effects across more than one disorder. Expression of *BTN2A2* was causal for MDD and SCZ, and *MRPL2* was causal for INS and SCZ, while that of other genes (for example, *ACTR5* and *NMB*) was causal for BIP and SCZ. It is also worth noting that the gene located within cg196244444 mentioned earlier, *MAD1L1*, is also a causal gene for SCZ. Further considering eQTL effects in 11 brain tissues, we found that among the causal genes identified in the IMAGEN sample, 28 were also causal for neuropsychiatric disorders through their differential expression in brain tissues (Supplementary Table 3b).

### Characterization of identified QTLs, CpGs and genes

**Fig. 5:**
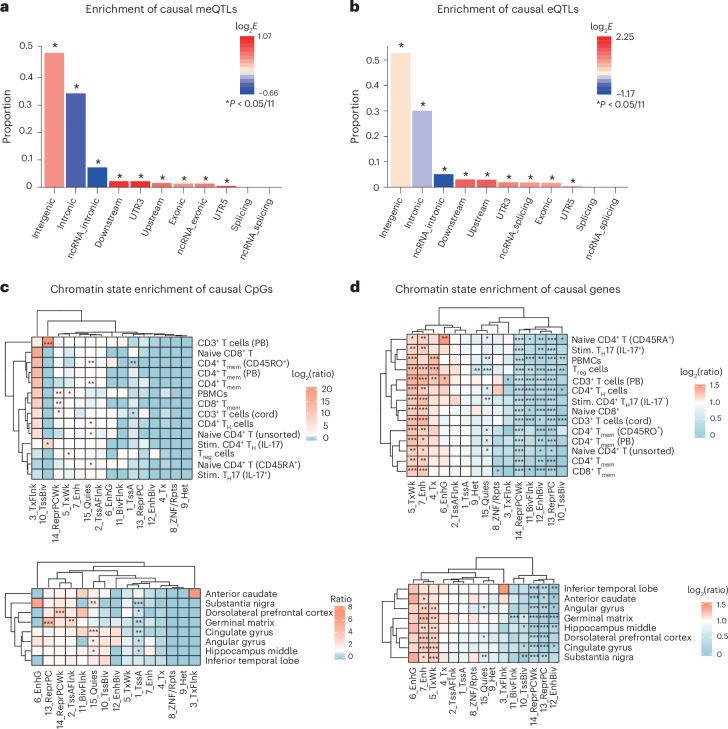
Functional characterization of identified causal CpGs and genes. **a**,**b**, Enrichment analysis of meQTLs and eQTLS mapped to causal CpGs and causal genes. Both causal *cis*-meQTLs (**a**) and *cis*-eQTLs (**b**) were enriched in intergenic regions while depleted in intronic regions (all *P* < 1 × 10^−16^). A two-sided hypergeometric test was used, with the Bonferroni-corrected significance threshold provided. **c**,**d**, Chromatin state enrichment of the causal CpGs (**c**) and genes (**d**) across blood and brain tissues, based on the core 15-state ChromHMM model defined by the NIH Roadmap Epigenomics Mapping Consortium. A two-sided hypergeometric test was used, with the Benjamini–Hochberg FDR method used for multiple testing correction. *E*, fold enrichment; BivFlnk, flanking bivalent transcription start sites and enhancer; CD3^+^ T cells (cord), primary CD3^+^ T cells from cord blood; CD3^+^ T cells (PB), primary CD3^+^ T cells from peripheral blood; CD4^+^ T_H_ cells, primary CD4^+^ T helper cells from peripheral blood (CD25^−^); CD4^+^ T_mem_ (PB), primary CD4^+^ effector/memory T cells from peripheral blood (CD25^int^, CD127^+^); Enh, enhancers; EnhBiv, bivalent enhancer; EnhG, genic enhancers; Het, heterochromatin; naive CD4^+^ T (CD45RA^+^), primary naive CD4^+^ T helper cells; ncRNA, non-coding RNA; PBMCs, peripheral blood mononuclear primary cells; Quies, quiescent or low activity; ReprPC, repressed polycomb; ReprPCWk, weak repressed polycomb; stim., stimulated; T_reg_ cells, primary CD4^+^ regulatory T cells from peripheral blood (CD25^+^, CD127^−^); TssA, active transcription start sites; TssAFlnk, flanking active transcription start sites; TssBiv, bivalent/poised transcription start sites; Tx, strong transcription; TxFlnk, transcription at 5′ and 3′ gene ends; TxWk, weak transcription; UTR, untranslated region; ZNF/Rpts, zinc finger genes and repeats.

To explore biological mechanisms underlying the SNP–disease associations, we conducted positional, tissue-specific and pathway enrichment analyses on the meQTLs (*N* = 34,758) and eQTLs (*N* = 14,360) mapped to putatively causal CpGs and genes, respectively. As shown in Fig. 5a,b, positional enrichment analyses indicated that both causal *cis*-meQTLs and *cis*-eQTLs were enriched in intergenic regions and depleted in intronic regions (fold enrichment_meQTLs_ = 1.34 and 0.75, fold enrichment_eQTLs_ = 1.13 and 0.83, all *P* < 1 × 10^−16^), consistent with previous findings that intergenic regions often harbor regulatory elements^16,17^.

To further characterize the regulatory landscape, we assessed chromatin state enrichment using the 15-state ChromHMM model from the Roadmap Epigenomics Project^39^ across brain and blood tissues (Supplementary Table 4). Causal CpGs (*n* = 85; Supplementary Table 2a) were significantly enriched in bivalent promoter regions in primary T cells from peripheral blood—a chromatin state associated with transcriptional silencing of key developmental genes, while maintaining them in a poised state for activation. In brain tissues, these CpGs were enriched in quiescent chromatin and depleted from actively transcribed regions, suggesting a role in developmentally regulated or repressed epigenetic contexts (Fig. 5c). By contrast, causal genes (*n* = 70; Supplementary Table 3a) showed strong and consistent enrichment across tissues in active regulatory states, including enhancers and regions of ongoing transcription, and were depleted in repressive chromatin states (Fig. 5d). These findings support the role of causal genes in dynamic transcriptional regulation relevant to neuropsychiatric risk.

Chromosome 6 (chr. 6p21 and chr. 6p22) was significantly enriched for causal CpGs, while chromosome 3 (chr. 3p21) was significantly enriched for causal genes (Supplementary Fig. 1). Causal genes tended to be expressed at significantly lower levels in several brain regions (for example, anterior cingulate, basal ganglia, amygdala, hippocampus, hypothalamus and cortex) and in the pancreas and liver (Supplementary Fig. 2). Unsurprisingly, causal genes and genes mapping to causal CpGs were significantly enriched for genes identified in GWAS for ASD, SCZ and BIP. They were also significantly enriched for genes implicated in diabetes and autoimmune diseases, such as ulcerative colitis and asthma (Supplementary Figs. 3–5), suggesting potential overlap between the genomic architecture of autoimmune diseases and neuropsychiatric disorders.

### Multi-omics integrative regulatory pathways for neuropsychiatric disorders

To further explore potential regulatory mechanisms underlying neuropsychiatric disorders, we conducted two additional analyses. First, we performed two-step MR analyses to identify causal genes mediating the effects of causal CpGs on these disorders. This analysis revealed 30 causal CpG–gene pairs, with 6 genes (that is, *PGBD1*, *ATXN1*, *HLA-DRB1*, *MRPL2*, *NT5DC1* and *MAD1L1*) identified as key mediators (Supplementary Table 5). Notably, certain CpG sites were found to exert causal effects through multiple genes. For instance, decreased expression of *MRPL2* mediated the effects of cg06770790 methylation on both INS and SCZ, whereas reduced *NT5DC1* expression mediated the effect of this CpG methylation on BIP. In addition, six CpGs causal for SCZ (cg00330953, cg10095777, cg13187827, cg19593741, cg23287992 and cg25264948) were linked to opposing regulatory effects on two genes: increasing *HLA-DRB1* expression while simultaneously decreasing expression of *PGBD1*. The proportion of effect mediated via these causal genes ranged between 12.7% and 89.3% (Supplementary Table 6).

**Fig. 6:**
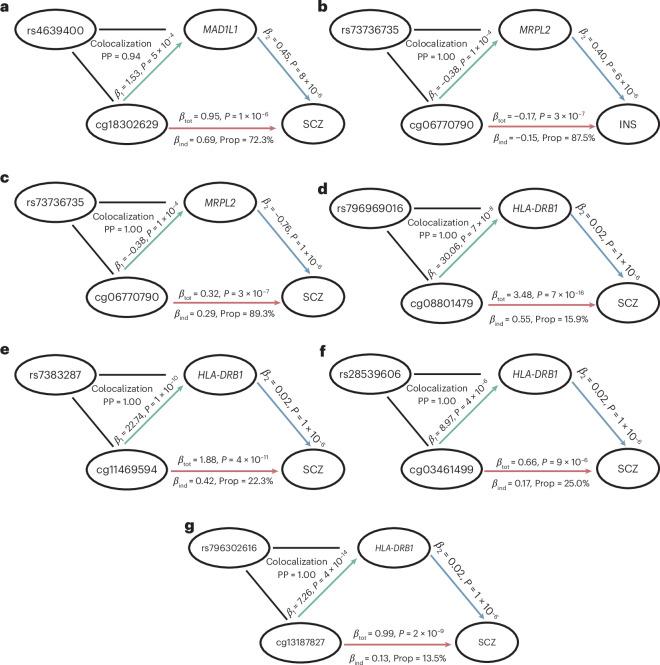
Multi-omics-integrated regulatory pathways for selected neuropsychiatric disorders. Two-step MR and colocalization analyses were conducted to reveal the regulatory pathways shared by multiple neuropsychiatric disorders. First, the colocalization analysis was conducted to identify CpG–gene pairs that share causal variants. Next, the indirect effect of CpG methylation on each neuropsychiatric disorder through gene expression (*β*_ind_) was calculated by multiplying the effect of CpG methylation on gene expression (*β*_1_) and the effect of gene expression on neuropsychiatric disorder (*β*_2_). The proportion mediated (Prop) was calculated by dividing *β*_ind_ by the total effect of CpG methylation on neuropsychiatric disorder (*β*_tot_). **a**, *MAD1L1* expression mediated the effect of cg18302629 methylation on SCZ. **b**,**c**, *MRPL2* expression mediated the effects of cg06770790 on both INS (**b**) and SCZ (**c**), indicating possible shared regulatory mechanisms for INS and SCZ. **d**–**g**, *HLD-DRB1* plays an important role in the regulatory mechanisms for SCZ by mediating the methylation of four CpG sites: cg08801479 (**d**), cg11469594 (**e**), cg03461499 (**f**) and cg13187827 (**g**). PP, posterior probability.

Next, we performed colocalization analyses on the 30 CpG–gene pairs identified above to determine if the neuropsychiatric disorders and their biological mediators (that is, DNA methylation and mRNA expression) shared common causal genetic variants. Our findings revealed strong evidence of colocalization (posterior probability >0.94) for six CpG–gene pairs associated with INS or SCZ (Fig. 6 and Supplementary Table 7). For SCZ, a causal meQTL (rs4639400, associated with the methylation of cg18302629) colocalized with an eQTL that increased *MAD1L1* expression (Fig. 6a). In addition, four causal meQTLs (rs796969016, rs7383287, rs28539606 and rs796302616, associated with the methylation of cg08801479, cg11469594, cg03461499 and cg13187827, respectively) colocalized with eQTLs that influenced *HLA-DRB1* expression (Fig. 6d–g). Moreover, a causal meQTL (rs73736735, associated with decreased cg06770790 methylation) colocalized with a causal eQTL associated with increased *MRPL2* expression (Fig. 6b,c). Importantly, both cg06770790 methylation and *MRPL2* expression were found to be causal for both INS and SCZ.

To assess the robustness of our findings, we reran key MR analyses using updated GWAS datasets for ADHD, BIP and MDD^14,40,41^. The results (Supplementary Table 8a,b) were largely consistent with the original analyses, with overlapping CpG sites and similar effect directions. Notably, the increased power of the updated MDD GWAS led to the identification of 32 putatively causal genes—substantially more than the 7 genes detected using the earlier dataset. Despite these additions, the core mechanisms highlighted in Fig. 6 remained unchanged, reinforcing the stability of our conclusions.

## Discussion

Our study offers a comprehensive resource of meQTLs and eQTLs in adolescent populations, providing a valuable platform to explore the multi-omic mechanisms underlying diseases, particularly those emerging in late childhood and early adolescence. Using a large-scale multicenter adolescent cohort, we identified over 9 million *cis*-meQTLs and 550,000 *cis*-eQTLs, more than double the numbers reported in previous adolescent studies^33^. These QTLs were validated in two independent datasets, demonstrating strong concordance in allelic effect directions upon replication. By leveraging this resource to investigate casual disease mechanisms across six neuropsychiatric disorders, our MR analyses identified causal CpG sites and genes, either unique to or shared among disorders. Combined with colocalization analyses, our findings uncovered potential transdiagnostic regulatory mechanisms. Notably, the expression of three genes—*MAD1L1*, *MRPL2* and *HLA-DRB1*—was shown to mediate the causal effects of CpG methylation on SCZ and INS. Specifically, decreased *MRPL2* expression due to methylation at cg06770790 was causal for both INS and SCZ, while increased expression of *MAD1L1* and *HLA-DRB1*, driven by methylation at several CpG sites (cg18302629, cg11469594, cg08801479, cg13187827 and cg03461499), was linked to SCZ. These findings provide crucial insights into key regulatory mechanisms and genes involved in neuropsychiatric disorders, highlighting potential therapeutic targets.

Several studies have previously linked DNA methylation to common mental disorders^42–52^, including candidate gene studies from postmortem human brain tissues^50,53^. Our study, however, goes beyond existing research by identifying causal CpGs using accessible tissues such as blood. Most of the causal CpGs we identified had not been found in previous epigenome-wide association studies^54^ (https://ngdc.cncb.ac.cn/ewas), with only a minority (33.3%; 4 of 12) having been reported in epigenome-wide association studies for MDD^55^, whereas none have been reported for other disorders. This difference likely stems from our focus on identifying causal, rather than downstream, effects of DNA methylation on disorders. In addition, the majority of existing epigenome-wide association studies are based on adult populations, with limited studies focusing on adolescents, largely due to the scarcity of large-scale datasets.

Interestingly, 13.25% of the causal meQTLs identified in our study achieved genome-wide significance in GWAS of corresponding disorders, underscoring the relevance of DNA methylation to disease risk. Among the CpGs identified, cg19624444 in *MAD1L1* was particularly noteworthy, showing opposite directionality across disorders: hypermethylation in blood associated with ADHD and MDD, and hypomethylation with BIP and SCZ. The tissue-specific effects are consistent with previous reports^38^, although some discrepancies between blood and brain findings warrant further investigation^37,56^.

Our eQTL-based analyses also validated a substantial number of genes previously reported in transcriptome-wide association studies^57^ (https://ngdc.cncb.ac.cn/twas/), with 40.0% (28 of 70) detected in blood transcriptome-wide association studies and 61.4% (43 of 70) across all tissues, confirming the robustness of our findings. Causal CpGs showed enrichment in the major histocompatibility complex region on chromosome 6 (chr. 6p21–p22), a known immune hotspot, while causal genes were notably enriched on chromosome 3p2. These genes also showed downregulation in several brain regions (for example, putamen, anterior cingulate, hippocampus and cortex) and in peripheral tissues, including pancreas, blood and liver. Moreover, causal CpGs and genes were significantly enriched for GWAS loci associated not only with ASD, SCZ and BIP but also with autoimmune diseases, such as type 1 diabetes, ulcerative colitis and asthma, supporting a potential shared immune–neuropsychiatric genetic architecture^27,58–61^. Chromatin state enrichment analyses further indicated that causal CpGs were significantly enriched in bivalent promoter regions in primary T cells—epigenetic regions associated with silenced but poised transcription of lineage-specific genes—and in quiescent chromatin regions in the brain. This pattern suggests that these CpGs may lie in developmentally regulated or immune-responsive regulatory elements. By contrast, causal genes showed consistent enrichment in active chromatin states (for example, enhancers and transcriptionally active regions) across both brain and blood, reinforcing their potential involvement in dynamic transcriptional programs relevant to neuropsychiatric and immune-related pathways. Both causal *cis*-meQTLs and *cis*-eQTLs were enriched in intergenic regions and depleted in intronic regions, consistent with previous work showing that intergenic regions are often involved in transcriptional regulation, while intronic regions are more commonly associated with post-transcriptional processes such as alternative splicing^16,17^. These distinct functional roles may have implications for how genetic variation influences gene regulation and may help prioritize regulatory elements as potential therapeutic targets. This regulatory localization aligns with the observed chromatin state enrichments and supports the functional relevance of the identified variants.

Several genes, such as *BTN2A2* and *NMB*, were transdiagnostic and showed differential expression, suggesting complex roles in both immune and neural systems. For example, *BTN2A2*, which was causal for MDD and SCZ and showed differential expression across blood and brain—and was previously found to be overexpressed in the hippocampus of patients with SCZ^62^—is known to modulate T cell-mediated immune responses^63–65^. By contrast, *NMB* was causal for both BIP and SCZ, with effects mediated by increased expression in blood and brain tissues. However, the varying expression profile of this gene across developmental stages and tissues may also contribute to these disorders^66^.

Further mechanistic insights emerged from our integrated genotype–DNA methylation–gene expression analyses. For example, both INS and SCZ were linked to elevated *MRPL2* expression, driven by reduced methylation at cg06770790. *MRPL2* encodes a mitochondrial ribosomal protein, supporting a role for mitochondrial dysfunction in psychiatric disorders^67^. Increased expression of *MAD1L1* and *HLA-DRB1*, driven by CpG methylation, was also causal for SCZ. *MAD1L1* has previously been linked to SCZ in GWAS^60,68,69^ and DNA methylation studies^37^. Human and animal models have shown that this gene, a component of the mitotic spindle-assembly checkpoint, is involved in neuronal migration and neurite outgrowth, thereby contributing to SCZ pathology^70^. Similarly, *HLA-DRB1*, part of the HLA complex involved in immune system regulation and a region whose differential methylation has been linked to multiple diseases, including rheumatoid arthritis, cancer, psoriasis and multiple sclerosis^71–75^, is implicated in disease pathology. Together, these findings highlight converging roles for mitochondrial function, neurodevelopmental processes and immune regulation in the epigenetic architecture of SCZ and INS, underscoring the potential of integrated multi-omic approaches to uncover biologically meaningful and therapeutically relevant mechanisms in psychiatric disorders.

The absence of detectable transdiagnostic CpG–gene mediation effects in disorders other than SCZ and INS likely reflects a combination of biological, methodological and technical limitations. Several disorders (for example, BIP, ADHD and ASD) are associated with smaller GWAS sample sizes, lower SNP-based heritability and weaker polygenic signals^76^, reducing statistical power. High clinical and genetic heterogeneity, particularly in MDD^77,78^, may obscure consistent regulatory pathways and affect MR precision. In some disorders, environmental influences may contribute more prominently to epigenetic architecture, limiting the sensitivity of genetically anchored instruments (for example, mQTLs and eQTLs). The smaller sample size of the gene expression dataset limited the identification of independent *cis*-eQTLs, necessitating a more permissive approach to IV selection in MR analyses. This may introduce bias, and future studies with larger transcriptomic cohorts are needed to validate these findings and refine the implicated regulatory networks. Crucially, MR-based inference relies on the assumption that genetic instruments affect the outcome solely via the exposure (for example, DNA methylation). This can be violated in the presence of horizontal pleiotropy, such as when *cis*-meQTLs alter transcription factor binding or gene expression independently of methylation. Although we applied MR-Egger regression to test for pleiotropy and excluded loci with significant intercepts, its limited power, especially with few instruments, means that pleiotropic effects cannot be ruled out. As such, we refer to identified CpGs and genes as putatively causal and stress the need for experimental validation. Further, our two-step MR approach assumes linear mediation from DNA methylation to gene expression to disease, which may not fully capture complex or non-transcriptional mechanisms. The use of *cis*-instruments in both steps also raises the possibility of pleiotropic effects due to LD. Our reliance on blood-based data and age mismatches between adolescent QTLs and adult GWAS may also reduce sensitivity, particularly for early-onset or brain-specific phenotypes. In addition, although we adjusted for immune cell composition using the first two principal components of estimated cell-type proportions, residual confounding may remain, particularly in relation to enrichments for immune-related signatures. Future studies using single-cell or sorted-cell data will be important to disentangle cell-intrinsic effects from composition-driven signals.

Despite these limitations, our study addresses an important gap by uncovering new insights into the epigenetic and transcriptomic regulation of neuropsychiatric disorders during adolescence, a critical developmental window for disease onset. Our findings highlight key genes and regulatory pathways, particularly those implicated in SCZ and INS, that may serve as promising targets for future therapeutic development across a broader spectrum of neuropsychiatric conditions.

## Methods

### DNA methylation microarray processing and normalization

Blood DNA methylation was assessed in IMAGEN using the Illumina HumanMethylation450 (450k) microarray, which measures CpG methylation across >485,000 probes covering 99% of the RefSeq gene promoters^79^, following the manufacturer’s protocols. Quality control and quantile normalization were performed in R using the minfi Bioconductor package^80^. Briefly, red and green channel intensities were mapped to the methylated and unmethylated status, and average intensities were used to check for low quality samples. Initial quality assessment was performed using the preprocessIllumina option. Principal component analyses were performed using the singular value decomposition method to identify methylation outliers based on the first four components. Samples with intensities more than 3 s.d. away from the median were considered outliers and removed. Intensities from the sex chromosomes were used to predict sex, and samples with discordant predicted and recorded sex were removed. Samples that were initially processed in batches were merged at this stage before further preprocessing. Stratified quantile normalization was then applied across samples. The data were then normalized together using the minfi preprocessQuantile function^81^. Principal component analyses of normalized *β* values were used to control for unknown structure in the methylation data. Cell counts for the six major cell types in blood (granulocytes, B cells, CD4^+^ T cells, CD8^+^ T cells, monocytes and NK cells) were estimated by implementing the estimateCellCounts function in minfi, which gives sample-specific estimates of cell proportions based on reference information on cell-specific methylation signatures. Analyses involving DNA methylation data were controlled for the first four principal components of the methylation data, sample batches, the first two components of estimated cell-type proportion, recruitment centers (dummy coded) and sex as covariates.

### mRNA processing

Gene expression was investigated in 631 participants from the IMAGEN sample for which mRNA expression data were available at the age of 14 (ref. ^82^). Total RNA was extracted from whole blood cells using the PAXgene Blood RNA Kit and labeled complementary RNA was prepared using the Illumina TotalPrep RNA Amplification kit. Gene expression profiling was performed by Illumina HumanHT-12 v4 Expression BeadChips (Illumina). Expression data were normalized using the mloess method^83^ and log transformed before analyses. Analyses involving gene expression data were controlled for recruitment centers (dummy coded) and sex as covariates.

### SNP genotyping

Genotypes were assessed in IMAGEN using two genotyping arrays. A total of 705 and 1,382 individuals were genotyped using the Illumina Human610-Quad BeadChip and the Illumina Human660-Quad BeadChip arrays, respectively, yielding genotype data for 582,982 markers. Quality control procedures were applied separately for each platform. SNPs were excluded if they had a call rate below 95%, a minor allele frequency less than 5%, significant deviation from Hardy–Weinberg equilibrium (*P* ≤ 1 × 10^−3^) or were non-autosomal. Individuals with a genotype failure rate exceeding 5% were also excluded. Population homogeneity was assessed using Structure software with HapMap reference populations^84^, and individuals with divergent ancestry (that is, not clustering with Utah residents of northern and western Europe descent) were removed. In addition, identity-by-state clustering and multidimensional scaling were performed using PLINK 1.90 to detect and exclude closely related individuals. Principal component analysis was then applied to further identify and remove outliers, defined as individuals located more than 4 s.d. from the mean on any of the first 20 principal components. After quality control, genotypes from both platforms were merged and platform-specific SNPs were excluded from subsequent analyses.

### *cis*-meQTL and *cis*-eQTL mapping

For *cis*-meQTL mapping in IMAGEN, a 3-step strategy was used due to the computational burden of running 5.9 million SNP × 374,000 CpG linear regression models. In the first step, residuals were obtained by regressing CpG methylation on all covariates, including age, sex, site, top two methylation principal components, wave, cell-type structure and genetic population structure. In the second step, linear regression was used to test the associations between residuals obtained in the first step and SNPs within a 2-Mb window of the corresponding CpG. During this step, candidate CpG–SNP pairs were prefiltered by a prefiltering *P* value threshold of 1 × 10^−6^. Finally, in the third step, associations between CpG methylation and SNPs for the candidate CpG–SNP pairs were recalculated by modeling CpG methylation as the dependent variable, SNP as the independent variable and adjusting for the same covariates in the first step, with a Bonferroni-corrected *P* value threshold 3.1 × 10^−11^ (0.05/1.6 × 10^9^ *cis*-CpG–SNP pairs).

For *cis*-eQTL mapping, we used a similar three-step strategy as used for *cis*-meQTL mapping. The prefiltering *P* value threshold was set to 1 × 10^−3^, and the final *P* value threshold was obtained using FDR correction, following previous studies^34^.

### Validation of *cis*-meQTLs and *cis*-eQTLs

Published studies^32,33^ of *cis*-meQTL summary statistics were used to validate our results of the *cis*-meQTL mapping. The KORA study included 3,799 participants of European ancestry and 3,195 participants of South Asian ancestry, with approximately 5 million and 9 million *cis*-meQTL–CpG pairs identified, respectively. The ARIES study included 837 adolescents of European ancestry and identified about 4 million *cis*-meQTL–CpG pairs. We used GTEx v8 *cis*-eQTL from whole blood samples^34^ to validate our *cis*-eQTL mapping results. GTEx v8 included 488 whole blood samples of European ancestry and identified about 1.6 million *cis*-eQTL–gene pairs for autosomes. A Bonferroni-corrected *P* value threshold was used to identify validated *cis*-meQTL–CpG pairs in KORA and ARIES, and a *P* value threshold based on FDR correction was used to identify validated *cis*-eQTL–gene pairs in GTEx v8.

In validating the *cis*-meQTLs and *cis*-eQTLs, we compared our own mapping results with the published results and calculated the coverage and consistency of the effect directions. Coverage is defined as the proportion of pairs in our mapping results that could also be found in the published study. To assess consistency of effect directions for these overlapping pairs, we calculated the proportion of *cis*-meQTLs with consistent effect directions (based on *β* values) on CpG methylation among the overlapping pairs.

### Identification of putatively causal CpGs and genes for neuropsychiatric disorders

To identify CpG sites and genes with putative causal effects on neuropsychiatric disorders, we applied a two-sample MR framework, in which genetic variants (IVs) associated with DNA methylation or gene expression were identified in the IMAGEN adolescent cohort (exposure dataset) and tested for association with disease outcomes using independent GWAS summary statistics (outcome dataset). This design minimizes confounding and reverse causation, as the instruments are defined independently of disease status. We used publicly available GWAS summary statistics for major psychiatric disorders, including the most comprehensive data available at the time of analysis, including: ADHD^85^, ASD^15^, BIP^10^, MDD^11^, SCZ^9^ and INS^13^. For ADHD, BIP and MDD, we incorporated summary data from the latest Psychiatric Genomics Consortium GWAS^14,40,41^, which became available during revision. Full details and data sources are listed in Supplementary Table 1.

We first mapped *cis*-meQTLs and *cis*-eQTLs in the IMAGEN adolescent cohort and pruned them for LD using a threshold of *r*^2^ < 0.01 within a 2-Mb window, based on the 1000 Genomes reference panel^86^. These LD-independent *cis*-QTLs were used as IVs to proxy methylation levels at CpG sites or expression of genes in the MR analyses. The two-sample MR design ensures that the exposure (methylation or expression) and the outcome (psychiatric disorder) are assessed in independent datasets, thereby reducing bias from reverse causation and residual confounding, as genetic variants are randomly allocated at conception.

A key assumption of MR is that the genetic instruments only influence the outcome through the exposure of interest (for example, CpG methylation or gene expression). However, this assumption may be violated by horizontal pleiotropy, in which a variant affects both the molecular trait and the disease through separate biological mechanisms. To account for this, we applied MR-Egger regression, which can detect and adjust for directional pleiotropy under the InSIDE (instrument strength independent of direct effect) assumption. We further evaluated consistency of causal effect estimates across multiple MR methods, including inverse variance weighted (IVW) and weighted median (WM) estimators. For CpGs, IVW and WM MR tests were performed only when at least three independent *cis*-meQTLs were available, which is the minimum required for multi-instrument MR. Where only one instrument was available, Wald ratio estimates were used. In the case of gene expression, we did not impose a minimum number of IVs due to the limited number of independent *cis*-eQTLs per gene. To assess the validity of MR estimates, we conducted heterogeneity tests and examined MR-Egger intercepts for evidence of directional pleiotropy.

A CpG site or gene was considered to have a significant putative causal effect on a given neuropsychiatric disorder if it satisfied the following criteria: (1) *P*_pleiotropy_ > 0.05 (no evidence of directional pleiotropy); (2) *P*_heterogeneity_ > 0.05 (no heterogeneity) and Bonferroni-corrected *P*_IVW_ < 0.05 (that is, *P*_IVW_ < 0.05 per number of tested CpGs or genes); or, if heterogeneity was present (*P*_heterogeneity_ < 0.05), then (3) Bonferroni-corrected *P*_WM_ < 0.05.

We emphasize that this statistical approach assumes the genetic instruments influence the outcome exclusively through the exposure (that is, DNA methylation or gene expression) and not through pleiotropic pathways. To account for potential violations of this assumption, we compared MR-Egger and IVW estimates and interpreted consistency in effect size and direction as supportive, although not definitive, evidence of robustness. All MR analyses were conducted using the TwoSampleMR 0.5.8 package in R^87^.

### Brain–blood methylation correlation

We interrogated a searchable DNA methylation database^88^ (https://epigenetics.essex.ac.uk/bloodbrain/) generated from matched DNA samples isolated from whole blood and 4 brain regions (prefrontal cortex, entorhinal cortex, superior temporal gyrus and cerebellum) from 122 individuals to establish the degree to which blood methylation levels at selected loci correlated with their brain methylation patterns. Correlations between blood and brain methylation levels at individual CpG sites were evaluated to indicate the similarity of methylation levels between blood and brain tissues at these sites.

### Functional enrichment analyses

SNP and gene-based enrichment analyses were conducted in FUMA^89^. The SNP2GENE option was used for enrichment analyses of xQTLs (that is, meQTLs and eQTLs) identified in this study.

The GENE2FUNC option was used to investigate potential biological pathways associated with causal genes and the genes closest to causal CpGs. Hypergeometric tests (with Bonferroni-corrected *P* value) were used to investigate over-representation of genes from multiple pathways. Analyses focused on pathway (KEGG), tissue (GTEx v8) and GWAS catalog-based enrichment analyses.

### Chromatin state enrichment analyses

To investigate the functional genomic context of CpGs and genes identified as putatively causal for psychiatric disorders, we performed chromatin state enrichment analyses using the National Institutes of Health (NIH) Roadmap Epigenomics Project’s 15-state ChromHMM model (https://egg2.wustl.edu/roadmap/web_portal/chr_state_learning.html#core_15state). This model integrates multiple histone modification marks to classify the genome into 15 discrete chromatin states, including active transcription start sites, enhancers, transcribed regions, polycomb-repressed domains and quiescent regions. Enrichment was performed separately for causal CpGs and causal genes across a panel of brain tissues (for example, hippocampus, anterior caudate and cingulate gyrus) and blood and T cell types (for example, peripheral blood mononuclear cells and CD4^+^ and CD8^+^ T cells). For CpG-level analyses, each causal CpG site was mapped to chromatin state annotations, and its overlap was assessed relative to a background of all tested CpG sites included in the meQTL analysis (*N* = 372,582). For gene-level analyses, each causal gene was mapped to its genomic span (transcription start to end site), and overlaps with chromatin states were compared with a background set of all genes tested in the eQTL analysis (*N* = 31,427). Enrichment ratios were calculated as the observed overlap divided by the expected overlap under a null model of random distribution. Statistical significance of enrichment was assessed using hypergeometric tests, and results were visualized as heat maps displaying enrichment ratios across tissues and chromatin states.

### Mediation of putatively causal CpGs effects on neuropsychiatric disorders via gene expression

Two-step MR analysis was conducted to assess the role of gene expression on the CpG–neuropsychiatric disorders causal pathway. In the first step, by using causal CpGs as the exposure, corresponding causal *cis*-meQTLs as the IVs and causal genes on the same chromosome as the outcome, we conducted MR analysis to assess the causal effect of CpGs on gene expression (*β*_1_), with Bonferroni-corrected *P* value thresholds. For example, 7 causal CpGs and 1 causal gene on chromosome 6 were identified for BIP in the previous MR analysis; therefore, the *P* value threshold for the first step MR of BIP was set as 0.05/(1 × 7). In the second step, for significant CpG–gene pairs identified in the first step, the causal effects of these causal genes to corresponding neuropsychiatric disorders (*β*_2_) were estimated via MR analysis, and mediation effects of CpG–neuropsychiatric disorder via gene expression were estimated by multiplying *β*_1_ and *β*_2_. Finally, proportion mediated via gene expression along the CpG–neuropsychiatric disorder was calculated by dividing mediation effect by the total effect, which is the causal effect of causal CpGs to corresponding disorders.

### Colocalization analysis

Colocalization analysis was performed on causal CpG–causal gene pairs identified by two-step MR analysis. Colocalization tests were performed on each CpG–gene pair using corresponding *cis*-meQTLs and *cis*-eQTLs. A Bayesian colocalization method implemented in the R package coloc 5.2.3 was used to test the probability of one SNP being causal for both the CpG and gene expression^90^. In this study, the association results between SNPs and CpGs (at *P* < 3.1 × 10^−11^) and between SNPs and gene expression (at FDR-corrected *P* < 0.05) within a 2-Mb window were used as input. Prior probabilities were set as suggested by previous studies^36^: the probability of a SNP being associated with trait 1 only was 2 × 10^−11^, the probability of a SNP being associated with trait 2 only was 1 × 10^−7^ and the probability of a SNP being associated with both traits was 2 × 10^−12^. A SNP was considered to be colocalized for a CpG and a gene if the posterior probability (PP4) was greater than 90%.

### Ethical statement

The IMAGEN study was approved by local ethnical research committees at each research site, including King’s College London (PNM/10/11-126), University of Nottingham (D/11/2007), Trinity College Dublin (SPREC092007-01), Technische Universitat Dresden (EK 235092007), Commissariat a l’Energie Atomique et aux Energies Alternatives, INSERM (2007-A00778-45), University Medical Center at the University of Hamburg (M-191/07) and in Germany by the medical ethics committee of the University of Heidelberg (2007-024N-MA) in accordance with the Declaration of Helsinki. In all studies, informed consent was sought from all participants and from a parent or guardian of each participant if under 18 years of age.

### Reporting summary

Further information on research design is available in the Nature Portfolio Reporting Summary linked to this article.

## Supplementary information

Supplementary Information Supplementary Figs. 1–5.

Reporting Summary

Supplementary Tables Supplementary Tables 1–8.

## Data Availability

All the primary results in this study can be found in the Supplementary Tables. The raw IMAGEN dataset is protected and not available due to data privacy laws. However, it may be obtained upon application by email via https://imagen-project.org/. Public QTL summary statistics from KORA, ARIES and GTEx studies are available as described at https://www.helmholtz-munich.de/en/epi/cohort/kora, http://www.ariesepigenomics.org.uk/ and https://gtexportal.org/home/, respectively. The correlation result between blood and brain methylation levels is available at https://epigenetics.essex.ac.uk/bloodbrain/. The links and references for all the publicly available summary statistics are as follows: ADHD GWAS, 10.6084/m9.figshare.14671965 (ref. ^91^) and 10.6084/m9.figshare.22564390 (ref. ^92^); ASD GWAS, 10.6084/m9.figshare.14671989 (ref. ^93^); BIP GWAS, 10.6084/m9.figshare.14102594 (ref.^94^) and 10.6084/m9.figshare.27216117 (ref. ^95^); MDD GWAS, https://datashare.ed.ac.uk/handle/10283/3203 and https://cncr.nl/research/summary_statistics/; INS GWAS, 10.6084/m9.figshare.19426775 (ref. ^96^); KORA meQTL, https://zenodo.org/record/5196216#.YRZ3TfJxeUk (ref. ^97^); ARIES meQTL, http://www.mqtldb.org; and GTEx v8 eQTL, www.gtexportal.org.

## Code availability

Primary analyses were conducted in R v.4.3.2. MR analyses were performed with the TwoSampleMR 0.5.8 package, and colocalization tests used the coloc 5.2.3 package. Custom code that supports the main findings of this study is available at https://github.com/qian40/causal_pathway_code and will also be available at Code Ocean (10.24433/CO.5785855.v1) upon publication.
