# Supplementary material for: Adolescent multi-omics and Mendelian randomization reveal transdiagnostic molecular mechanisms in psychiatric disorders
Source: Nat Ment Health. 2026 Jun 5;4(8):1248–61. doi: 10.1038/s44220-026-00660-2 (PMC13441900; doi:10.1038/s44220-026-00660-2)
Supplement: Supplementary file 1 [file 44220_2026_660_MOESM1_ESM.pdf]

nature mental health

Supplementary information https://doi.org/10.1038/s44220-026-00660-2

Adolescent multi-omics and Mendelian randomization reveal transdiagnostic molecular mechanisms in psychiatric disorders

In the format provided by the authors and unedited

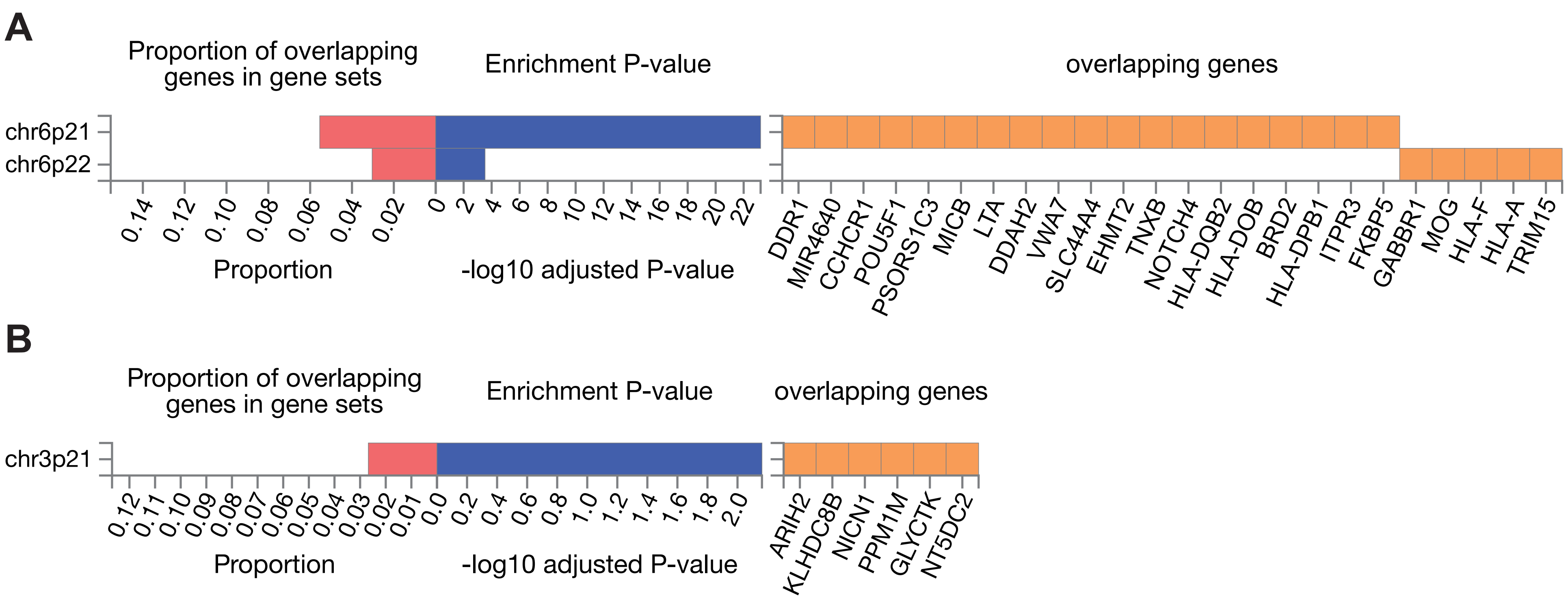

Supplementary Figure 1. Positional enrichment results for (A) nearest genes of causal CpGs (n=73 CpGs) and (B) causal genes (n=62 genes). Two-sided hypergeometric test was used, with Bonferroni method used for multiple correction.

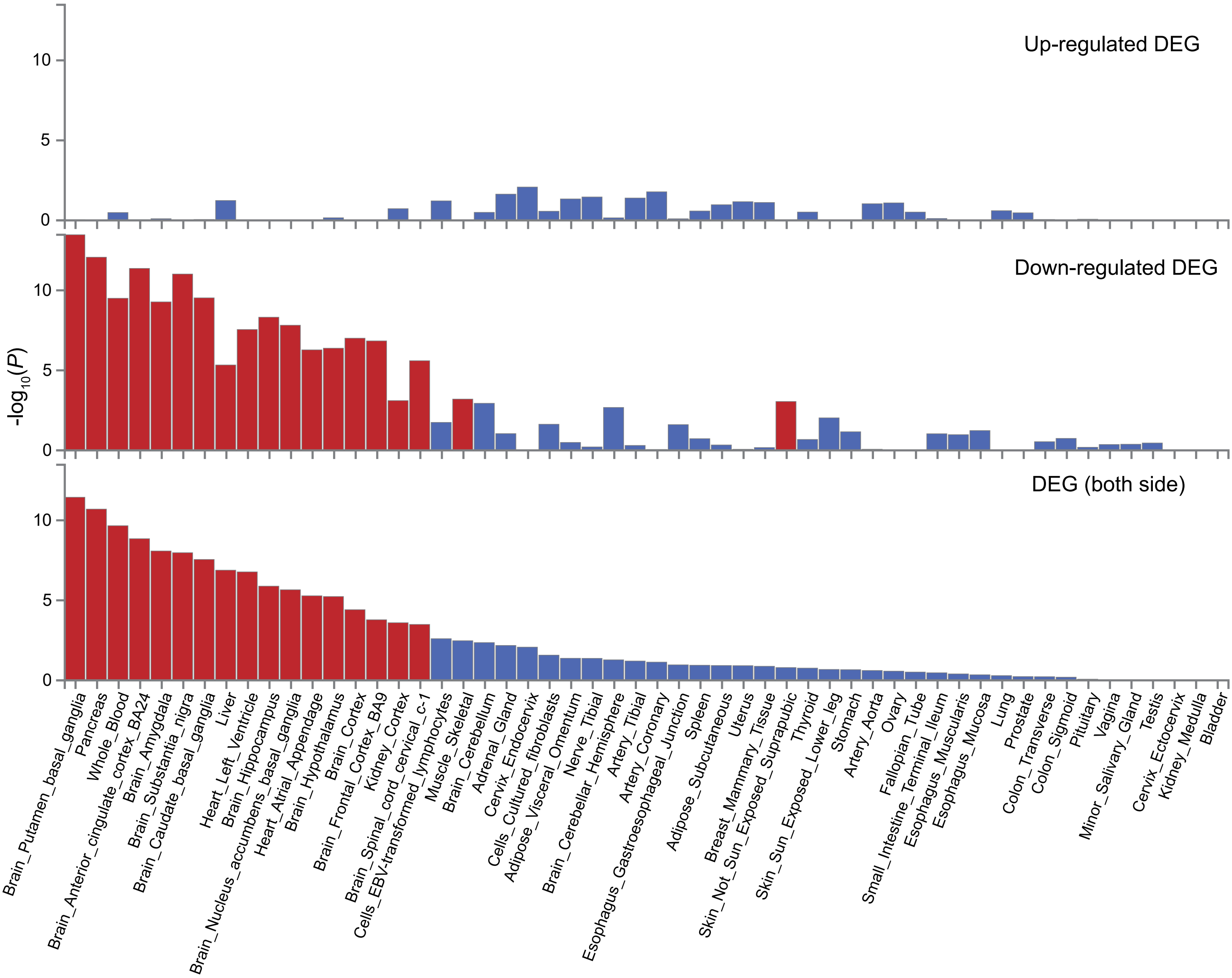

Supplementary Figure 2. Tissue expression results of causal genes (n=62 genes) on 53 specific tissue types by GTEx in the FUMA. Two-sided hypergeometric test was used, with Bonferroni method used for multiple correction.

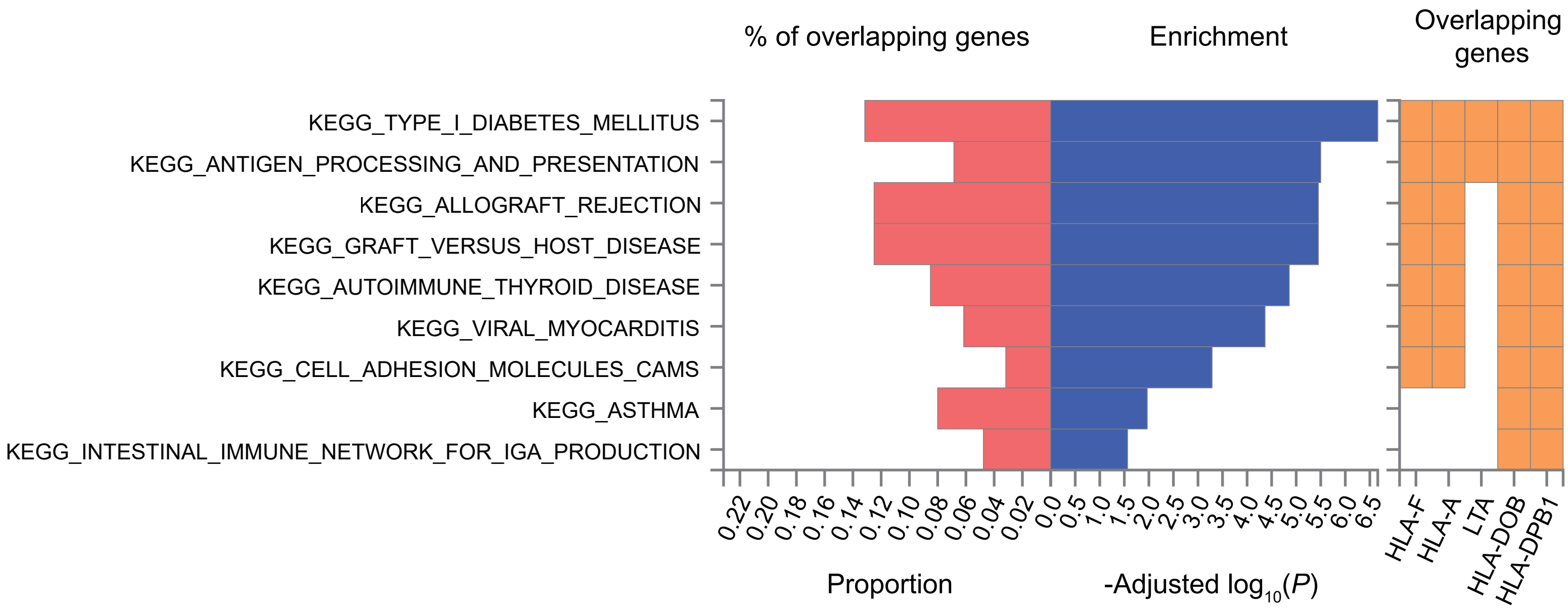

Supplementary Figure 3. KEGG pathway enrichment analysis of closest genes of causal CpGs (n=73 CpGs). Two-sided hypergeometric test was used, with Bonferroni method used for multiple correction.

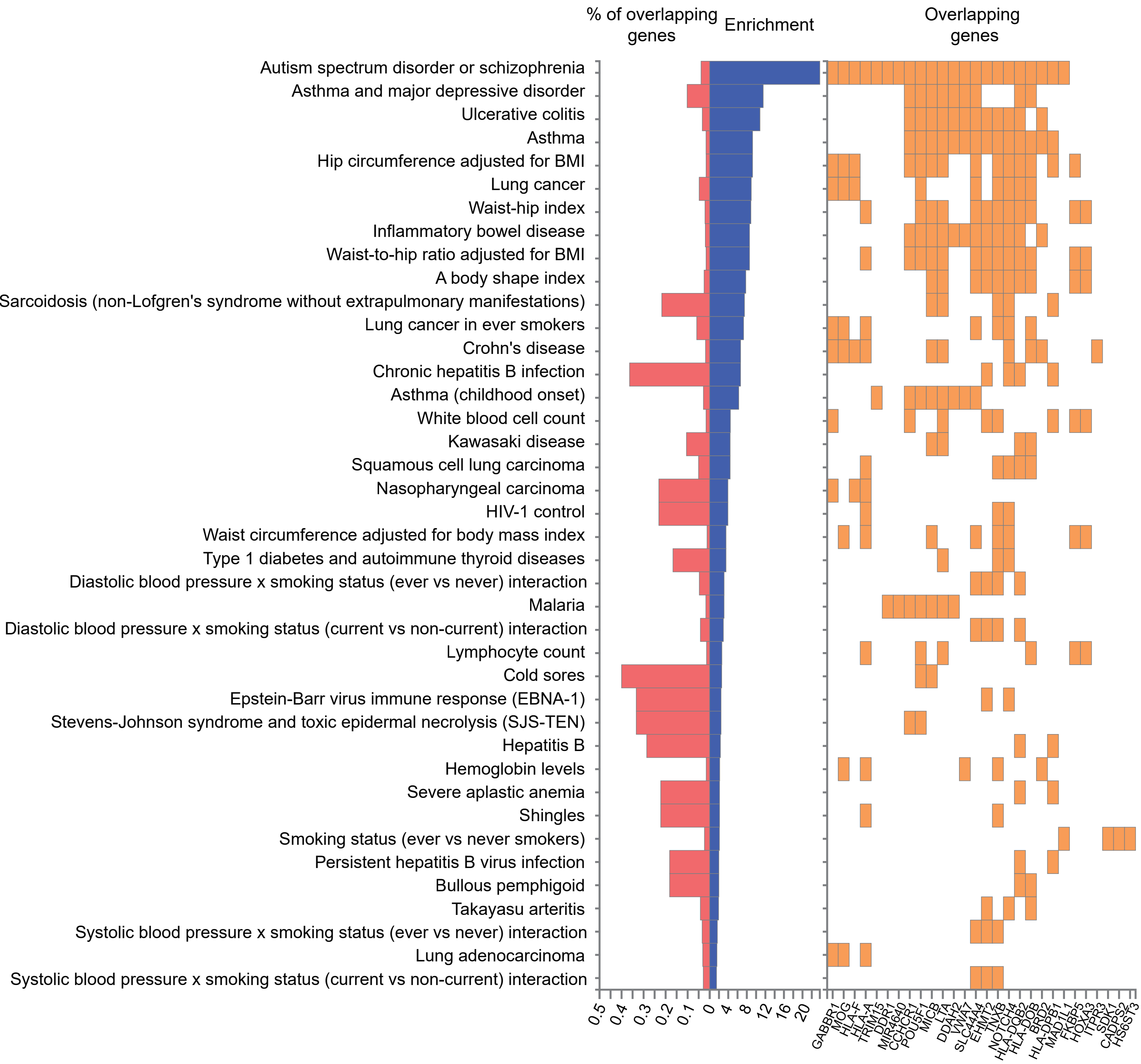

Supplementary Figure 4. Gene set enrichment analysis of closest genes of causal CpGs (n=73 CpGs). Two-sided hypergeometric test was used, with Bonferroni method used for multiple correction.

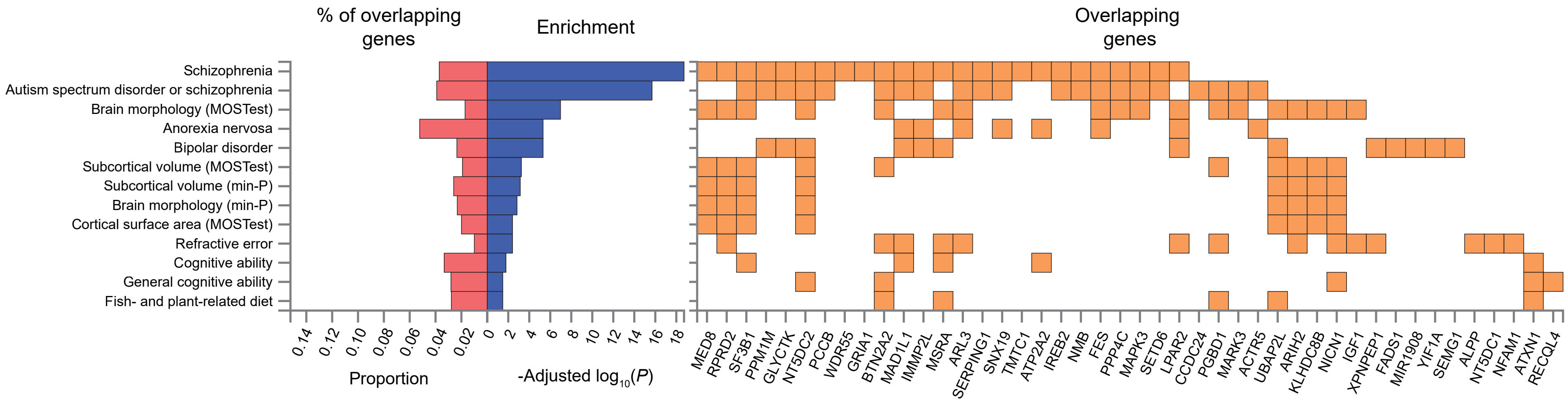

Supplementary Figure 5. Gene set enrichment analysis of causal genes (n=62 genes). Two-sided hypergeometric test was used, with Bonferroni method used for multiple correction.
